# Supplementary material for: Plastic response of Medicago sativa L. root system traits and cold resistance to simulated rainfall events
Source: PeerJ. 2021 Sep 9;9:e11962. doi: 10.7717/peerj.11962 (PMC8435203; doi:10.7717/peerj.11962)
Supplement: Supplemental Information 2 [file peerj-09-11962-s002.docx]

**Information on irrigation time and quota among three simulated rainfall events.**

|  | 1^st^ day | 2^nd^ day | 3^rd^ day | 4^th^ day | 5^th^ day | 6^th^ day | 7^th^ day | 8^th^ day |  |
| --- | --- | --- | --- | --- | --- | --- | --- | --- | --- |
| D_2_ |  | X_1_ |  | X_2_ |  | X_3_ |  | X_4_ | …… |
| D_4_ |  |  |  | X_1_+X_2_ |  |  |  | X_3_+X_4_ | …… |
| D_8_ |  |  |  |  |  |  |  | X_1_+X_2_+X_3_+X_4_ | …… |

Note: X_1_、X_2_…… and X_8_ represent different irrigation amounts.
