# Supplementary material for: Plastic response of Medicago sativa L. root system traits and cold resistance to simulated rainfall events
Source: PeerJ. 2021 Sep 9;9:e11962. doi: 10.7717/peerj.11962 (PMC8435203; doi:10.7717/peerj.11962)
Supplement: Supplemental Information 3 [file peerj-09-11962-s003.docx]

**Changes of soil relative water content under different irrigation frequencies. (%WHC)**

|  | 1^st^ day | 2^nd^ day | 3^rd^ day | 4^th^ day | 5^th^ day | 6^th^ day | 7^th^ day | 8^th^ day |  |
| --- | --- | --- | --- | --- | --- | --- | --- | --- | --- |
| D_2_ | 55.1 | 50.6 | 54.2 | 51.6 | 56.3 | 50.1 | 55.4 | 51.8 | …… |
| D_4_ | 56.3 | 51.1 | 47.7 | 41.1 | 57.1 | 52.5 | 46.3 | 42.1 | …… |
| D_8_ | 55.8 | 49.7 | 47.3 | 42.4 | 37.2 | 35.1 | 32.5 | 30.1 | …… |
