# Supplementary figures and images for: Plastic response of Medicago sativa L. root system traits and cold resistance to simulated rainfall events
Source: PeerJ. 2021 Sep 9;9:e11962. doi: 10.7717/peerj.11962 (PMC8435203; doi:10.7717/peerj.11962)

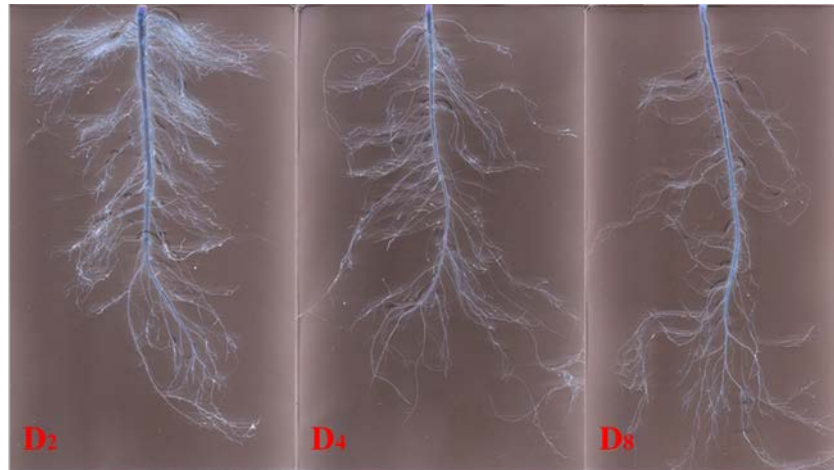

**Scanning image of root system under three simulated rainfall events.**

Supplement: Supplemental Information 4 [file peerj-09-11962-s004.pdf]
